# Molecular Dynamics and Experimental Investigation on Biological Properties of Polyetheretherketone/Graphene Oxide/Hydroxyapatite Composites

**DOI:** 10.3390/polym18141683

**Published:** 2026-07-08

**Authors:** Jin Liu, Long Chen, Ge Gao, Fei Ren, Yukui Cai, Zhanqiang Liu

**Affiliations:** 1 Key Laboratory of High Efficiency and Clean Mechanical Manufacture, Ministry of Education, Jinan 250061, China; liujinmech@mail.sdu.edu.cn (J.L.);; 2 School of Mechanical Engineering, Shandong University, Jinan 250061, China; 3 Ministry of Education Key Laboratory for Advanced Textile Composite Materials, Tiangong University, Tianjin 300387, China; 4 National Key Laboratory of New Pharmaceutical Preparations and Excipients, School of Pharmacy, Hebei Medical University, Shijiazhuang 050017, China; 5 Shandong Luxing Intelligent Technology Co., Ltd.; Jinan 250000, China

**Keywords:** polyetheretherketone, molecular dynamics simulation, hydroxyapatite, graphene oxide

## Abstract

Polyetheretherketone (PEEK) is a favorable material in bone tissue engineering due to its excellent mechanical properties and biocompatibility. However, as PEEK is biologically inert, this study introduced hydroxyapatite (HA) and graphene oxide (GO) to modify PEEK, and PEEK/GO/HA composites were prepared via compression molding and sintering. Molecular dynamics simulation results indicated that the Young’s modulus of the composite increased with rising HA content. The trends in the bulk modulus and shear modulus suggested a possible downward trend around HA contents of 10 wt% and 20 wt%; this may be attributed to the polarity mismatch between HA and PEEK, as well as the composite preparation process. The thermal conductivity of the composites exhibited a similar trend, with the thermal conductivity decreasing until the HA content reached 30 wt% due to interfacial thermal resistance between PEEK and HA. Concurrently, in vitro cell culture experiments were conducted on the precursor powder mixture to investigate the effect of the composition ratio on biological properties. The results indicated that cell viability was higher when the HA content was 30 wt%. This demonstrates the significant potential of PEEK/GO/HA composites in the field of bone tissue engineering.

## 1. Introduction

Severe bone defects cannot be healed by the body’s own regenerative capacity and must be repaired or replaced through external intervention in order to restore the structural integrity and physiological function of the bone; therefore, bone grafting is commonly utilized to repair bone defects [1,2]. The main strategies for clinical repair of bone defects include autologous bone grafting, allogeneic bone grafting, and the use of synthetic bone implants. However, autologous bone grafting is associated with drawbacks such as secondary damage to the donor site, limited availability, and postoperative complications, whereas allogeneic bone grafting presents risks of immune rejection and disease transmission [3]. Consequently, developing high-performance artificial bone implant materials to overcome the limitations of biological grafts represents a central focus in the field of bone tissue engineering. An ideal artificial bone implant should possess excellent biocompatibility, favorable mechanical properties matching those of natural bone, and the ability to remain stable over the long term within the complex in vivo environment [4,5].

For a long time, metallic materials, including titanium alloys and stainless steel, have been extensively employed in the fabrication of orthopedic internal fixation devices and joint replacement prostheses due to their high mechanical strength, excellent fatigue resistance, and mature processing technologies [6]. However, long-term clinical practice and research have revealed a number of shortcomings associated with these metallic implants. For instance, their excessively high elastic modulus can induce stress shielding, whereby the implant bears the majority of the load, whilst the surrounding bone atrophies due to insufficient stress stimulation. This may ultimately result in implant loosening, secondary fractures, and trigger inflammatory reactions and allergic responses [7].

Polymer composites have emerged as an alternative option owing to their favorable biocompatibility and tunable mechanical properties. Among these, polyetheretherketone (PEEK), a high-performance semi-crystalline thermoplastic polymer, has attracted extensive attention in the field of orthopedics as an ideal substitute for conventional metallic implants due to its outstanding overall performance. PEEK possesses remarkable mechanical properties and an elastic modulus comparable to that of human bone [8,9,10]. Although the elastic modulus of PEEK is lower than that of primary weight-bearing bones, it can be adjusted through approaches such as filler reinforcement. Furthermore, PEEK exhibits good biocompatibility [11]. The biocompatibility of materials is of paramount importance. Materials must not induce inflammatory reactions or exhibit immunogenicity or cytotoxicity, ensuring that they are non-toxic and non-allergenic when in contact with the human body [12,13]. Furthermore, PEEK is translucent to X-rays and does not produce artifacts in post-operative medical imaging, which facilitates the monitoring of the bone healing process [14]. However, the bio-inertness of PEEK results in poor osseointegration, thereby limiting its clinical application [15]. To impart positive bioactivity to PEEK whilst retaining its excellent intrinsic properties, this study employed a composite fabrication method to synergistically incorporate graphene oxide (GO) and hydroxyapatite (HA) into the PEEK matrix.

GO is an important graphene derivative. As a typical two-dimensional nanomaterial, it has a large specific surface area and shows great potential in the field of biomaterials [16,17]. The surface and edges of GO are rich in oxygen-containing functional groups such as hydroxyl, carbonyl, and carboxyl groups, offering significant potential for surface modification, improvement of matrix-reinforcement phase interactions, and an increase in GO hydrophilicity and dispersibility [18]. Moreover, GO exhibits excellent osteoconductive and osteoinductive abilities for regulating osteoblastic differentiation [19]. In terms of mechanical reinforcement, when GO is uniformly dispersed as a nanofiller within a polymer matrix, its oxygen-containing functional groups enable it to form interactions such as hydrogen bonds with the polymer. Also, the two-dimensional planar structure of GO provides geometric constraints, which can enhance the mechanical strength of the composite material [20].

Simultaneously, the bioactive component HA was incorporated to improve the bio-inertness of PEEK [21]. As a major component of bone tissue, hydroxyapatite can promote osteoblast growth and stimulate the formation of new bone cells [22]. Furthermore, the nanoscale dimension of HA significantly increases its surface area and reactivity [23]. Additionally, the introduction of HA can optimize the surface morphology of the composites, providing more favorable conditions for cell adhesion and proliferation. Consequently, compounding HA into the PEEK matrix can significantly enhance the potential of the material in bone tissue engineering.

This study employed a multi-scale research approach. At the theoretical simulation level, molecular dynamics simulations were conducted to investigate the thermal conduction behaviors and load-transfer mechanisms of GO and HA within the PEEK matrix, providing a theoretical foundation for understanding the macroscopic properties of the composites. At the material characterization level, the microstructure of the composites was analyzed using scanning electron microscopy. In addition, in vitro cell culture experiments were conducted on precursor powder mixtures to identify the material composition yielding the highest cell viability. This comprehensive research framework, encompassing computational simulations, material preparation, property characterization, and biological evaluation, aims to provide a solid theoretical and experimental foundation for the development of high-performance bone implant composites.

## 2. Molecular Dynamics Simulation

In this study, molecular dynamics simulations were employed to construct composite models and investigate their thermal conductivity and mechanical properties. The constructed graphene sheets were oxidized using hydroxyl and carboxyl functional groups to obtain graphene oxide models. The PEEK polymer models were constructed using PEEK monomers, with the degree of polymerization set to 10. Spherical nanoclusters with a radius of 5 Å were built based on the hydroxyapatite unit cell to simulate hydroxyapatite nanoparticles.

Based on the Monte Carlo algorithm, GO sheets, PEEK chains, and spherical HA nanoclusters were randomly packed into a periodic cubic lattice with an initial density of 0.8 g/cm^3^ and dimensions of approximately 45 Å × 45 Å × 45 Å, according to the target composite proportions, thereby constructing the initial model of the ternary composites. In this molecular dynamics simulation, the COMPASS III force field was selected for all simulations, which is suitable for the construction and calculation of material models [24,25].

To minimize system energy, geometry optimization of the initial composite models was performed using the Forcite module. Geometry optimization employs the Smart algorithm, which utilizes the most rapid descent method and the conjugate gradient method. The system was then annealed using an NPT system, which maintains a constant number of particles, pressure, and temperature, with initial velocities assigned randomly and a temperature control algorithm based on Berendsen’s method, resulting in models with lower energy and more reasonable structures.

Molecular dynamics simulations were then performed under NPT conditions, with the pressure maintained at 1 × 10^−2^ GPa and the temperature at 298 K. The time step was set to 1 fs, and the total simulation duration was 500 ps. Calculations of Van der Waals forces were performed using the Atom-Based method, while electrostatic interactions were handled by the Ewald summation method. Observation of the dynamics density curves indicated that the system density had essentially reached equilibrium. This simulation procedure corresponds to the “compression molding” stage of the manufacturing process. Under constant external pressure, the model’s volume will dynamically adjust, and its density will increase, thereby simulating the densification effect resulting from the compaction process.

**Figure 1:**
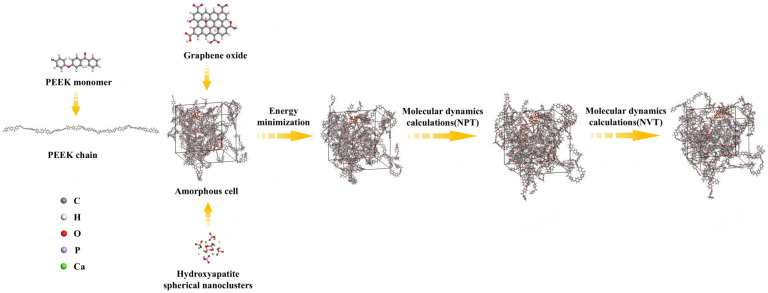
Model construction and simulation process in a molecular dynamics study.

Finally, in order to preserve the densification effect resulting from the previous compaction steps and to simulate the temperature during the preparation process, molecular dynamics simulations were carried out under the NVT system, maintaining a constant number of particles, volume, and temperature, with the temperature set at 663.15 K. The time step was set to 1 fs, and the total simulation duration was 1000 ps. Calculations of Van der Waals forces were performed using the Atom-Based method, while electrostatic interactions were handled by the Ewald summation method. This procedure ultimately simulated the structure of the composites following high-temperature sintering. The model construction and simulation process is shown in Figure 1.

### 2.1. Thermal Analysis

This study employed Perl code to calculate the thermal conductivity of the composites using the reverse non-equilibrium molecular dynamics (RNEMD) method. The principle can be explained as follows: during the simulation process, the model is divided into a number of layers of equal width along the direction of heat conduction, and ‘heat source layers’ and ‘cold source layers’ are defined. The simulation was conducted under the NVE system, wherein a velocity exchange operation was executed at regular time step intervals: the velocity vectors of the particle with the lowest kinetic energy in the hot layer and the particle with the highest kinetic energy in the cold layer were swapped. The kinetic energy transferred during a single exchange is denoted as ΔE. Over the total simulation time *t*, the cumulative exchanged energy is denoted as ΣΔE. The cross-sectional area A of the model, perpendicular to the direction of the heat flux, is given by $A=l_{x}\times l_{y}$. The formula for calculating the heat flux density *J* is as follows:

(1) $$J=\frac{\Sigma \Delta E}{2At}$$

The factor 2 in the denominator arises from the two heat flow paths formed by the periodic boundary conditions.

The spontaneous reverse heat flow generated within the system will gradually compensate for the artificially applied energy exchange. When the two reach equilibrium, the system establishes a stable temperature gradient and enters a steady state. In the steady state, a stable temperature gradient forms between the various layers.

According to the equipartition theorem, the local temperature of each layer is determined by the script using the kinetic energy of the enclosed atoms, as shown below.

(2) $$T=\frac{1}{3Nk_{B}}\sum_{i=1}^{N}m_{i}v_{i}^{2}$$

In this formula, $m_{i}$ and $v_{i}$ represent the mass and velocity of the *i*-th atom in the layer, respectively; N is the total number of atoms in that layer; and $k_{B}$ is the Boltzmann constant.

Upon achieving the steady-state temperature distribution, a least-squares linear fitting was performed on the temperature distribution within the linear response region to determine the temperature gradient dT/dz. According to Fourier’s law, the thermal conductivity is then calculated as follows:

(3) $$\lambda =-\frac{J}{dT/dz}$$

### 2.2. Mechanical Analysis

Using the Forcite module, the elastic mechanical properties of PEEK/GO/HA composites were predicted. The simulation process primarily consists of two stages: geometric optimization and the calculation of elastic constants. Prior to the elastic modulus calculations, geometry optimization was performed on the initial model using the Smart algorithm. Within the elastic range, the stress–strain relationship of a material follows a linear relationship described by the generalized Hooke’s law, as shown in the formula below:

(4) $$\sigma_{i}=C_{ij}\varepsilon_{j}$$

In the formula, $\sigma_{i}$ represents the stress vector, $\varepsilon_{j}$ represents the strain vector, and $C_{ij}$ represents the 6 × 6 elastic stiffness matrix.

In this study, the constant strain method was used to calculate $C_{ij}$. For the geometrically optimized unit cell, a set of small strains was applied along six independent strain directions, and the Vieri stresses of the system under each strain were then calculated.

The elastic constants $C_{ij}$ were derived through the linear fitting of the stress–strain data, and the compliance matrix $S_{ij}$ was obtained by calculating the inverse matrix of the elastic stiffness matrix:

(5) $$S_{ij}=C_{ij}^{-1}$$

Consequently, the Young’s modulus in different directions can be calculated; $E_{X}$, $E_{Y}$, and $E_{Z}$ represent the Young’s modulus in the directions of *x*, *y*, and *z*, respectively. The Young’s modulus can be calculated using the following formula.

(6) $$E_{X}=\frac{1}{S_{11}},E_{Y}=\frac{1}{S_{22}},E_{Z}=\frac{1}{S_{33}}$$

Calculate the average Young’s modulus, $E_{Avg}$, as an estimate of the overall Young’s modulus:

(7) $$E_{Avg}=\frac{1}{3}\left(E_{X}+E_{Y}+E_{Z}\right)$$

$E_{Max}$ is the maximum Young’s modulus in the three directions. The bulk modulus B and shear modulus G of the material were calculated according to the Voigt–Reuss–Hill theory. The Hill modulus is the average of the Voigt and Reuss moduli, providing a more effective modulus estimation.

The Voigt bulk modulus is calculated using the following equation:

(8) $$B_{V}=\frac{1}{9}\left[\left(C_{11}+C_{22}+C_{33}\right)+2\left(C_{12}+C_{13}+C_{23}\right)\right]$$

The Voigt shear modulus is calculated using the following equation:

(9) $$G_{V}=\frac{1}{15}\left[\left(C_{11}+C_{22}+C_{33}\right)-\left(C_{12}+C_{13}+C_{23}\right)\right]+\frac{1}{5}\left(C_{44}+C_{55}+C_{66}\right)$$

The Reuss bulk modulus is calculated using the following equation:

(10) $$B_{R}=\frac{1}{\left(S_{11}+S_{22}+S_{33}\right)+2\left(S_{12}+S_{13}+S_{23}\right)}$$

The Reuss shear modulus is calculated using the following equation:

(11) $$G_{R}=\frac{15}{4\left(S_{11}+S_{22}+S_{33}\right)-4\left(S_{12}+S_{13}+S_{23}\right)+3\left(S_{44}+S_{55}+S_{66}\right)}$$

The Hill modulus is calculated using the following equation:

(12) $$B_{H}=\frac{B_{V}+B_{R}}{2},G_{H}=\frac{G_{V}+G_{R}}{2}$$

Through the aforementioned molecular dynamics simulations, the elastic properties of PEEK/GO/HA composites with varying HA contents can be calculated. This enables us to clarify the relationship between different HA contents and the elastic modulus of the composites, offering key theoretical guidance for optimizing HA content in composite preparation.

## 3. Experimental Investigations

### 3.1. Materials

Graphene oxide was purchased from Suzhou Hengqiu Technology Co., Ltd., Suzhou, China with a purity exceeding 95%, consisting of 1–2 layers, a sheet diameter ranging from 10 to 50 µm, and a thickness of 0–1 nm. Hydroxyapatite was purchased from Shanghai McLean Biochemical Technology Co., Ltd., Shanghai, China with a particle size of 200 nm and a purity of 97%. Polyetheretherketone was purchased from Victrex, Lancashire, UK with a purity of 99% and a particle size of approximately 15 µm. Thiazol Blue MTT was purchased from Shanghai Yuanye Biotechnology Co., Ltd., Shanghai, China. DEME high-glucose medium was purchased from Wuhan Seville Biotechnology Co., Ltd., Wuhan, China.

### 3.2. Preparation Process

**Figure 2:**
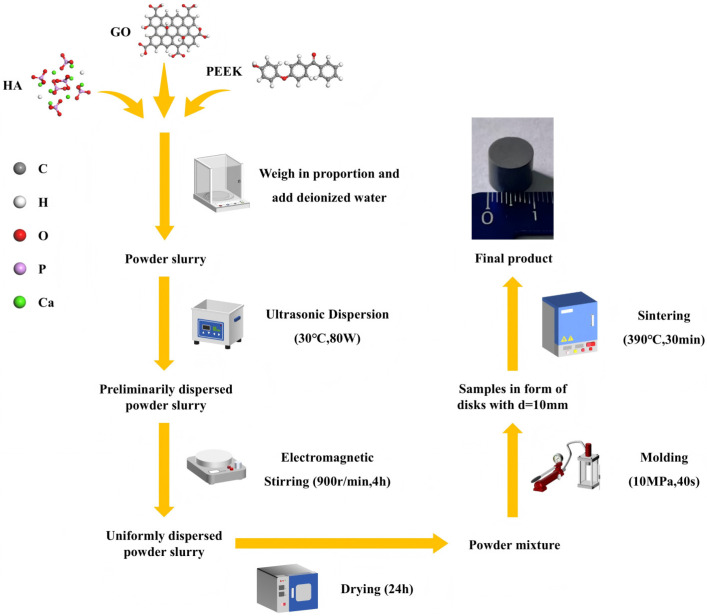
The preparation process of PEEK/GO/HA composites.

GO, PEEK, and HA powders were added into separate beakers in the proportions shown in Table 1. Deionized water was then introduced, and the mixture was subjected to ultrasonic agitation at 30 °C and 80 W for 40 min. Magnetic stirring at 900 rpm for more than 4 h was then applied to ensure the uniform dispersion of GO, PEEK, and HA. The beaker was then placed in a drying oven and dried for more than 24 h to obtain the dry mixed powder. The powder was spread into a cylindrical mold and compacted in a tablet press at a pressure of 10 MPa for 40 s to form a dense GO/PEEK/HA composite sheet. Finally, the resulting GO/PEEK/HA composite sheet was placed in a mold, wrapped in aluminum foil, and the mold was then placed in a box-type resistance furnace. Sintering was performed at 390 °C for 30 min, and upon cooling, the final dense GO/PEEK/HA composite was obtained. The samples were prepared in the form of disks with a diameter of 10 mm and a height of approximately 6 mm. Table 1 shows the densities of the five batches of composite materials prepared. The preparation process for the PEEK/GO/HA composite material is shown in Figure 2:

### 3.3. Microscopic Evaluation of Composites

Scanning electron microscopy (FE-SEM, Sigma 360, Carl Zeiss, Jena, Germany) was used to examine the surface of the samples and to analyze the distribution of the various components. Prior to SEM examination, the samples were sputter-coated with gold and subsequently scanned at an accelerating voltage of 3 kV to observe the surface morphology of the PEEK/GO/HA composites and the distribution of hydroxyapatite particles. ImageJ 1.54d, a piece of software that can be used for image analysis, was utilized to evaluate pore size and porosity to assist in analyzing the impact of hydroxyapatite incorporation and variations in its content on the pore structure of the composite.

### 3.4. Cell Viability Study

In this study, human umbilical vein endothelial cells (HUVECs) were purchased from Wuhan Punose Life Technology Co., Ltd., Wuhan, China. HUVECs were used to conduct in vitro experiments on a mixture of PEEK/GO/HA precursor powders to investigate the effect of the composition ratio on biological properties.

HUVECs were cultured in DMEM medium supplemented with 10% fetal bovine serum, 100 μg/mL streptomycin, and 100 IU/mL penicillin. The cells were maintained at 37 °C in a humidified environment containing 5% CO_2_. Once the HUVECs reached 85–95% confluence, trypsinization was performed with a solution containing 0.2% trypsin, 0.02% EDTA, and 0.05% glucose. This detachment process enabled us to harvest the cells from the culture flasks and subsequently prepare a diluted cell suspension to achieve the desired cell concentration.

The HUVECs were seeded into a 96-well plate at a density of 1 × 10^4^ cells per well and cultured in medium for 1 day. The samples were sterilized to ensure the removal of potential contaminants and subsequently suspended in culture medium. Then 10 μL of the sample powder suspension was added to the wells containing the cell suspension, and the plates were incubated in a 37 °C, 5% CO_2_ incubator for 24 h.

**Figure 3:**
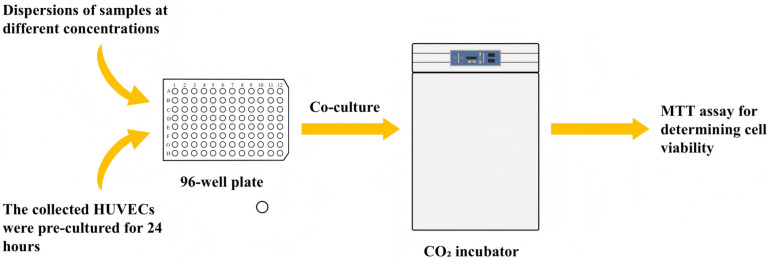
Experimental procedure for determining cell viability using the MTT assay.

Cell viability of HUVECs cultured with the sample dispersion for 24 h under standard conditions was assessed using the MTT assay. The experimental procedure is shown in Figure 3.

## 4. Result and Discussions

### 4.1. SEM Analysis

**Figure 4:**
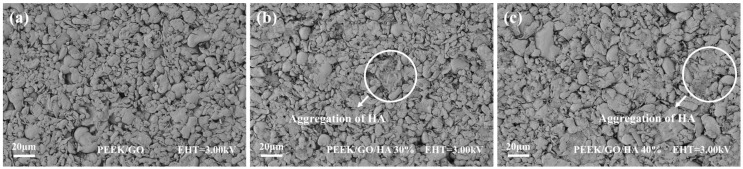
(**a**) SEM image of the PEEK/GO composite; (**b**) SEM image of the PEEK/GO/HA 30 wt% composite; (**c**) SEM image of the PEEK/GO/HA 40 wt% composite.

Figure 4 shows SEM images illustrating the surface morphology of the PEEK/GO/HA composite. As can be seen from Figure 4a–c, due to the incorporation of hydroxyapatite, the PEEK/GO/HA 30 wt% and PEEK/GO/HA 40 wt% composites exhibit a rougher surface compared to the PEEK/GO group, which is conducive to cell adhesion, spreading, and proliferation.

Furthermore, the SEM results presented in Figure 4b,c reveal a distinct distribution profile of hydroxyapatite nanoparticles within the PEEK/GO/HA 30 wt% and 40 wt% composites. The labeled sections in the figure revealed the aggregation of hydroxyapatite, which is primarily concentrated in the microscopic grooves on the sample surface. The average pore size and porosity of the composites, determined using ImageJ software, are shown in Table 2.

Due to the increased incorporation of hydroxyapatite and the preparation process, both the average pore size and porosity of the composites showed a decreasing trend, which may affect the adhesion and proliferation of cells on the surface of the composites.

Conducting SEM analysis of the PEEK/GO/HA composites is crucial for understanding their structural characteristics and for exploring innovative methods to fabricate composites for biomedical applications, thereby helping to advance their use in the field of bone tissue engineering.

### 4.2. Thermal Conductivity Analysis

**Figure 5:**
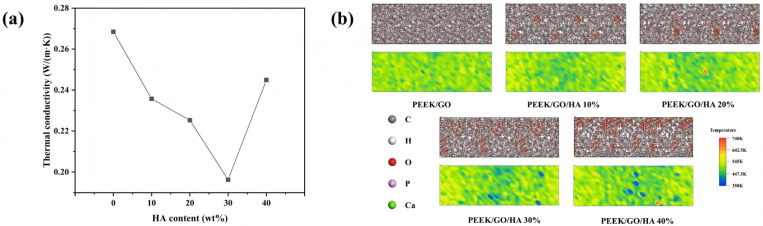
(**a**) Thermal conductivity of the composites. (**b**) Models of the composites and temperature distribution diagrams.

As shown in Figure 5a, the results of the molecular dynamics simulation indicated that, when the HA content was below 30%, the thermal conductivity decreased as the HA content increased. As shown in Figure 5b, the temperature distribution map of the model revealed areas of significant temperature differences at the edges of the HA, indicating a high interfacial thermal resistance between the HA and PEEK [26,27].

As the HA content increased, the total interfacial area between PEEK and HA within the system increased. The negative impact on the thermal conductivity of the composites caused by interfacial thermal resistance resulting from the increased HA content outweighed the advantage of the high thermal conductivity of HA, ultimately manifesting macroscopically as a reduction in the thermal conductivity of the composites.

As the HA content continued to increase, the thermal conductivity rebounded. This may be due to increased HA content, whereby HA aggregated and contributed to the formation of new thermal conduction pathways. Although interfacial thermal resistance still existed, the thermal conduction pathways formed by HA began to dominate, leading to an increase in thermal conductivity.

Consequently, the incorporation of HA holds great potential for modulating the thermal conductivity of the composites, enabling the composites to respond more effectively to changes in ambient temperature. This facilitates the rapid establishment of thermal equilibrium between bone implant materials and surrounding tissues, thereby preventing temperature fluctuations from interfering with the bone healing process.

### 4.3. Mechanical Properties

Based on a set of results of the molecular dynamics simulations, as shown in Table 3, it can be observed that the Young’s modulus of the composite material increases with rising HA content. The value of $E_{Max}$ showed an upward trend until the HA content reached 30 wt%, followed by a slight decrease at 40 wt%. This phenomenon can be attributed to the influence of the directionality of the components within the model. As the HA content increased from 0 wt% to 40 wt%, the value of $E_{Avg}$ showed a consistent upward trend; when the HA content reached 40 wt%, the Young’s modulus of the composites reached its maximum, representing a 43.3% increase compared to the group with 0 wt% HA.

The trend in the bulk modulus of PEEK/GO/HA composites differs from the monotonic increase observed in the Young’s modulus. Prior to an HA content of 20 wt%, the bulk modulus of the composites decreased as the HA content increased; however, once the HA content reached 20 wt%, the bulk modulus began to rise again with increasing HA content.

The shear modulus of the PEEK/GO/HA composite exhibited a more complex trend. The incorporation of 10 wt% HA increased the shear modulus of the PEEK/GO/HA composites. However, when the HA content reached 20 wt%, the shear modulus decreased, falling even below that of the PEEK/GO group. Once the HA content reached 20 wt%, the shear modulus of the composite material can be approximated as increasing with rising HA content. When the HA content was 30 or 40 wt%, the shear modulus of the composites was significantly enhanced, increasing by more than 55% compared to the PEEK/GO group.

**Figure 6:**
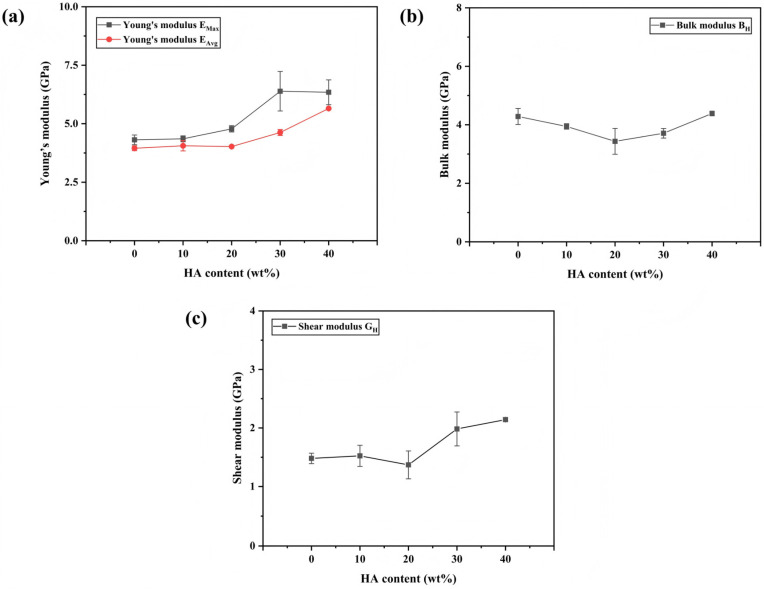
Mechanical properties obtained from molecular dynamics simulations: (**a**) Young’s modulus, (**b**) bulk modulus, (**c**) shear modulus.

As shown in Figure 6a, the increasing trend of the Young’s modulus of the PEEK/GO/HA composites with rising HA content is consistent with the findings of related studies [28,29]. This phenomenon can be attributed to the high stiffness of HA restricting the movement of PEEK chains; as the HA content increased, the Young’s modulus of the composites increased [30].

**Figure 7:**
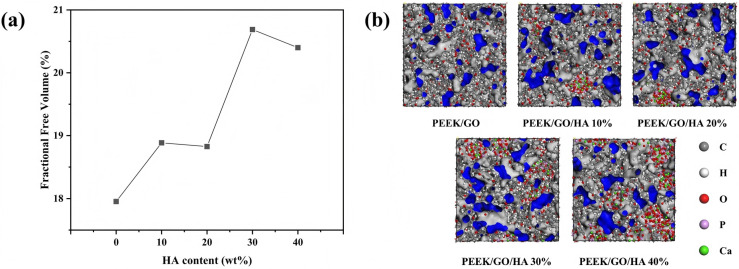
(**a**) Fractional free volume of the composite models. (**b**) Volume visualization of the composite models.

The bulk modulus of PEEK/GO/HA composites exhibited a decreasing trend until the HA content reached 20 wt%. This may be related to the preparation process. As shown in Figure 7a, the fractional free volume of the composite models can be considered to increase with the ascending HA content. This indicates that the microscopic pores in the composites increase as the HA content increases. The pressure of 10 MPa caused the composite to form a relatively dense structure, whereas the addition of small amounts of HA may lead to the formation of microscopic pores due to the mismatch in polarity between HA and PEEK. As shown in Figure 7b, there are microscopic pores between the PEEK and the HA. These pores will be compressed under pressure, resulting in a reduction in the bulk modulus of the material. Once the HA content reached 20 wt%, the bulk modulus of the composites increased. This is likely due to the formation of a rigid load-bearing network as the HA content rises, which effectively bears the stress and resists compression, resulting in a recovery of the bulk modulus that continues to increase as the HA content rises [31].

The shear modulus of PEEK/GO/HA composites increased when the HA content was low; this can be explained by the fact that HA restricts the movement of the PEEK chains. However, the shear modulus of PEEK/GO/HA composites reached a minimum when the HA content reached 20 wt%. This is likely due to a polarity mismatch between PEEK and HA.

When the HA content reached a high level, more microscopic pores were introduced, making slippage between the hydroxyapatite nanoparticles and the PEEK chains more likely to occur, which manifests as a reduction in the shear modulus of the composites. When the HA content exceeded 20 wt%, the shear modulus can be considered to increase as the HA content rose. This is likely because the HA forms a rigid load-bearing network that effectively absorbs stress, resulting in an increase in the shear modulus of the composites.

### 4.4. Cell Viability Study by MTT Assay

**Figure 8:**
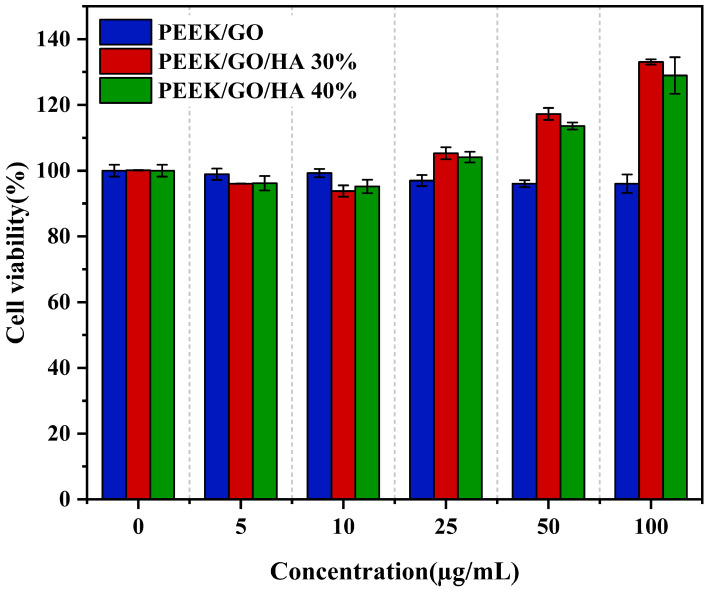
Bar chart showing cell survival rates.

The results of the cell viability assay are shown in Figure 8:

The cell viability of the PEEK/GO group was slightly higher than that of the PEEK/GO/HA 30% and PEEK/GO/HA 40% groups when the sample concentration was below 10 μg/mL. This may be due to the lower concentration of HA at this stage, which limits its effectiveness in promoting cell survival and proliferation. When the sample concentration exceeded 25 μg/mL, it was observed that cell viability in the PEEK/GO/HA 30% and PEEK/GO/HA 40% groups was significantly higher than in the PEEK/GO group. At the same time, the PEEK/GO/HA 30% group exhibited the highest cell viability, which is highly consistent with results from relevant studies; this indicates that the composition ratio is a key factor determining the biological performance of this composite system [32]. Furthermore, the cell viability of the PEEK/GO/HA 30% group and the PEEK/GO/HA 40% group exceeded 100%, demonstrating their ability to promote cell proliferation. Meanwhile, the increased content of the bioactive material HA can promote cell proliferation through the release of bioactive ions such as calcium and phosphorus, as well as the presence of hydrophilic groups on the surfaces of GO and HA [33,34]. Composites containing HA did not exhibit significant cytotoxicity. Furthermore, some studies suggest that the incorporation of GO into such composites can confer antimicrobial properties on the material [35].

It was also found that the cell viability of the PEEK/GO/HA 30% group was higher than that of the PEEK/GO/HA 40% group, providing important formulation guidance for the design of composites with controllable properties.

## 5. Conclusions

In this study, PEEK/GO/HA composites with varying proportions were successfully prepared using the compression molding and sintering method. Molecular dynamics simulations were also conducted to investigate the thermal conduction behavior and load transfer mechanisms of the composites at different HA contents.

The HA content has a significant effect on the thermal conductivity of the composites. When the HA content is below 30 wt%, the thermal conductivity of the composites decreases as the HA content increases; above this level, the thermal conductivity of the composites increases as the HA content rises. Different levels of HA incorporation exhibit complex effects on the elastic moduli of the composites. Specifically, the Young’s modulus of the composites increases continuously with higher HA contents. For the bulk modulus, when the HA content is less than 20 wt%, the bulk modulus decreases as the HA content increases; beyond this content, the bulk modulus increases as the HA content rises. With regard to the shear modulus, a lower content of HA enhances the shear modulus of the composites; however, at 20 wt%, the shear modulus of the composites drops to its lowest value. Beyond this concentration, the shear modulus of the composites can be considered to increase again as the HA content rises. Due to the incorporation of HA, the surface of the composite exhibits a higher degree of roughness, which has a positive effect on cell proliferation. In vitro cell culture studies have shown that cell viability in both the PEEK/GO/HA 30% group and the PEEK/GO/HA 40% group was higher than in the PEEK/GO group without HA. Furthermore, cell viability was highest in the PEEK/GO/HA 30% group.

Overall, these findings suggest that PEEK/GO/HA composites hold great promise for applications in bone tissue engineering.

## Figures and Tables

**Table 1:** Composition and proportions of composite materials.

Serial Number	Sample	PEEK (wt%)	GO (wt%)	HA (wt%)	Density (g/cm^3^)
1.	PEEK/GO	95	5	-	1.052
2.	PEEK/GO/HA 10%	85	5	10	1.114
3.	PEEK/GO/HA 20%	75	5	20	1.144
4.	PEEK/GO/HA 30%	65	5	30	1.159
5.	PEEK/GO/HA 40%	55	5	40	1.259

**Table 2:** Average pore size and porosity calculated from the SEM results of the samples.

Sample	Average Pore Size (µm)	Porosity (%)
PEEK/GO	1.303 ± 0.061	4.813 ± 0.777
PEEK/GO/HA 30%	1.022 ± 0.062	4.079 ± 0.439
PEEK/GO/HA 40%	1.000 ± 0.075	3.691 ± 0.481

**Table 3:** Mechanical property parameters of MD simulations (GPa).

Sample	E_X_	E_Y_	E_Z_	E_Max_	E_Avg_	B_H_	G_H_
PEEK/GO	4.072	4.013	3.669	4.072	3.918	4.460	1.379
PEEK/GO/HA 10%	4.212	3.559	4.034	4.212	3.935	3.865	1.657
PEEK/GO/HA 20%	4.709	3.390	4.070	4.709	4.056	3.005	1.229
PEEK/GO/HA 30%	7.240	4.030	3.910	7.240	5.060	3.795	2.280
PEEK/GO/HA 40%	5.693	5.416	5.730	5.730	5.613	4.435	2.174

## Data Availability

The raw data supporting the conclusions of this article will be made available by the authors on request.
